# Consistent detection of *Trypanosoma brucei* but not *T. congolense* DNA in faeces of experimentally infected cattle

**DOI:** 10.1038/s41598-024-54857-5

**Published:** 2024-02-20

**Authors:** Isabel Saldanha, Martha Betson, Christina Vrettou, Edith Paxton, James Nixon, Peter Tennant, Adrian Ritchie, Keith R. Matthews, Liam J. Morrison, Stephen J. Torr, Lucas J. Cunningham

**Affiliations:** 1 Vector Biology Department, Liverpool School of Tropical Medicine, Liverpool, UK; 2 School of Veterinary Medicine, University of Surrey, Guildford, UK; 3 Roslin Institute, University of Edinburgh, Edinburgh, UK; 4 Large Animal Research and Imaging Facility, University of Edinburgh, Edinburgh, UK; 5 Institute of Immunology and Infection, University of Edinburgh, Edinburgh, UK; 6 Department of Tropical Disease Biology, Liverpool School of Tropical Medicine, Liverpool, UK

**Keywords:** Parasite biology, Parasite host response, Infectious-disease diagnostics, Pathogens, PCR-based techniques

## Abstract

Animal African trypanosomiasis (AAT) is a significant food security and economic burden in sub-Saharan Africa. Current AAT empirical and immunodiagnostic surveillance tools suffer from poor sensitivity and specificity, with blood sampling requiring animal restraint and trained personnel. Faecal sampling could increase sampling accessibility, scale, and species range. Therefore, this study assessed feasibility of detecting *Trypanosoma* DNA in the faeces of experimentally-infected cattle. Holstein–Friesian calves were inoculated with *Trypanosoma brucei brucei* AnTat 1.1 (n = 5) or *T. congolense* Savannah IL3000 (n = 6) in separate studies. Faecal and blood samples were collected concurrently over 10 weeks and screened using species-specific PCR and qPCR assays. *T. brucei* DNA was detected in 85% of post-inoculation (PI) faecal samples (n = 114/134) by qPCR and 50% by PCR between 4 and 66 days PI. However, *T. congolense* DNA was detected in just 3.4% (n = 5/145) of PI faecal samples by qPCR, and none by PCR. These results confirm the ability to consistently detect *T. brucei* DNA, but not *T. congolense* DNA, in infected cattle faeces. This disparity may derive from the differences in *Trypanosoma* species tissue distribution and/or extravasation. Therefore, whilst faeces are a promising substrate to screen for *T. brucei* infection, blood sampling is required to detect *T. congolense* in cattle.

## Introduction

Species of *Trypanosoma* transmitted by the tsetse fly (*Glossina*) vector are responsible for clinically significant disease in both human and animal populations. Although substantial in-roads have been made in the control of human African trypanosomiasis (HAT), with disease transmission targeted for elimination by 2030^1^, animal African trypanosomiasis (AAT) remains a disease of significant economic burden in sub-Saharan Africa. AAT is the cause of significant morbidity and mortality in susceptible ruminant hosts, impacting food security, productivity and livelihoods for associated human populations^2,3^.

Three key salivarian *Trypanosoma* species are responsible for AAT; *Trypanosoma brucei* sensu-lato (of the subgenera *Trypanozoon*), *T. congolense* (of *Nannomonas*) and *T. vivax* (of *Duttonella*)^4^. In terms of pathogenesis, *T. brucei* has the ability to extravascularly invade mammalian tissue in addition to populating blood and lymph^5^, whilst available evidence suggests *T. congolense* Savannah is restricted to the circulatory system, including by adherence to endothelial cells^6–8^. Susceptible hosts include domestic cattle, sheep, goats, equids, dogs, in addition to many wildlife species^3,9,10^. Although most *Trypanosoma* infections in wildlife species are asymptomatic, wildlife hosts play a significant role in transmission and maintenance of AAT at the wildlife-livestock interface^10,11^. In domestic animals, the disease is characterised by a broad range of pathologies including acute anaemia, weight loss, abortion, loss of body condition, and eventually death if left untreated^9^. Aside from mortality, production loss can come in the form of reduced milk and meat yields, reduced fertility, reduced ability of animals to perform agricultural work, and decreased market value of affected animals. Through these direct and indirect impacts, AAT is estimated to be responsible for a combined US$4.5 billion in agricultural losses every year^2^.

There are no pathognomonic clinical signs unique to AAT, with symptoms mirroring those of multiple co-endemic diseases present in the region, such as anaplasmosis and babesiosis^12,13^. Therefore, despite empirical diagnosis being the most commonly used method, both sensitivity and specificity is poor. Direct observation of parasites in blood or lymph and immunodiagnostic assays in particular have poor applicability in remote field settings^12–14^. Molecular tools have greatly improved the sensitivity and specificity of AAT diagnosis, however high cost and limited pen-side suitability remain a barrier for use in affected regions^12,14^. These obstacles make accurate diagnosis-led treatment of AAT an ongoing challenge. The widespread use and misuse of trypanocide chemotherapy is likely to have played a role in the emergence of trypanocide resistance^15^, which has been well-documented in several AAT-endemic regions^15,16^. Therefore, there is an urgent need to explore alternative AAT diagnostic methods.

Being able to accurately detect and characterise *Trypanosoma* species in livestock hosts is also key for disease surveillance of both HAT and AAT. Rhodesian HAT (rHAT), caused by *T. brucei rhodesiense*, is a zoonotic disease with livestock and wildlife reservoirs. Therefore, surveillance in animal hosts can aid in disease mapping, increase accuracy of disease modelling, and inform the use of trypanocide use or vector controls measures at a region-wide scale. A range of different molecular detection methods have been developed and used over the years, including DNA probes and PCR and restriction fragment length polymorphism (RFLP)^12^. For the molecular detection of both *T. brucei* sensu-lato and *T. congolense* in livestock clinical samples*,* molecular targets have included species-specific satellite DNA repeat sequences that have been designed for *T. brucei* s-l and *T. congolense* Savannah, Kilifi and Forest subtypes^17^, the internal-transcribed spacer 1 (ITS1) region and associated rDNA that amplifies all species/strains, and a recently described small RNA (7SLRNA) target secreted by *Trypanosoma* during active infection^18,19^. Of these, the minichromosomal satellite 177 bp DNA tandem repeat regions are the most sensitive targets, with copy number estimated at 10,000 per cell in *T. brucei* s-l^20^. Although the 177 bp *T. brucei* repeat (TBR) target locus was recently confirmed to be more heterogeneous than initially anticipated^21^, it remains the most sensitive and widely-used molecular target in the form of TBR-PCR, SYBR green TBR-qPCR and a novel probe-based TBR-qPCR assay^17,21–23^. Similarly, the 316 bp *T. congolense* Savannah repeat region (TCS) remains a long-established and widely used target, developed as both TCS-PCR and SYBR-based TCS-qPCR^17,20,22^. However, confirmation of *T. brucei rhodesiense* presence is still reliant on the detection of the single-copy serum resistance-associated (SRA) gene, which continues to present a major challenge in assay sensitivity^24^.

Using faeces as a diagnostic tool, otherwise known as ‘copro-diagnostics’, is not a novel concept. For decades, faecal matter has been used as a diagnostic and surveillance tool for a wide variety of pathogens, from antimicrobial resistant bacteria in cattle to COVID-19 in humans^25–27^. Although identifying genetic markers of blood-borne protozoa in faeces would appear unlikely, there have even been several successful studies in humans and wildlife, including detection of *Plasmodium sp.*, *Leishmania sp.* and *Trypanosoma sp.*^28–33^. The method by which *Trypanosoma* DNA enters the faeces is not known but is likely to involve gastrointestinal tract (GIT) pathology. GIT pathology findings in *T. b. brucei* experimentally-infected mice have included villus atrophy and oedema of the lamina propria as a result of inflammation^34,35^. Although extravascular *T. brucei* parasites have been found in the peritoneal fluid of experimentally-infected cattle^36^ and the adipose tissue and lymph nodes surrounding the mesentery of infected mice^37,38^, *T. brucei* has not been recorded in the GIT mucosa or lumen. In contrast, we are not aware of any evidence of *T. congolense* in these locations.

A key pitfall of current molecular diagnostic use in cattle is the requirement for blood samples. Collecting blood samples requires veterinary-trained personnel, specialised equipment and the capture and restraint of individual cattle. Such methods are laborious, costly, carry risk of injury to the animal and personnel, and reduce capacity for representative herd sampling in AAT and HAT surveillance studies. These logistical challenges are even more acute for the sampling of AAT and rHAT wildlife hosts. Sampling faeces has the potential to remove these obstacles, thus presenting as an attractive option that can reduce cost and increase the scalability of sampling. *T. brucei* DNA detection has been successfully demonstrated in wild chimpanzees (*Pan troglodytes*) and experimentally-infected mice (*Mus musculus*) faeces, by using nested PCR targeting the *Trypanosoma*-specific ITS1 region, with species identification confirmed by sequencing of resultant amplicons^28^. However, to date there have been no studies carried out to assess the feasibility of detecting *Trypanosoma* DNA in livestock faeces, nor the detection of *T. congolense* DNA in faeces from any infected host.

Therefore, this study set out to assess the feasibility of detecting the DNA of two *Trypanosoma* species of veterinary importance (*T. brucei brucei* and *T. congolense* Savannah) in faecal samples derived from experimentally infected cattle, and to compare these results to those obtained from matched blood samples collected over 10-week longitudinal studies.

## Results

### qPCR assay optimisation

For both TBR-qPCR and TCS-qPCR, 59 °C was determined to be the optimal T_a_ (S1) on the basis of high sensitivity and maximised assay efficiency. Additionally for both assays, 400 nM primer and 200 nM probe optimal concentrations were determined to give optimal performance (S1). Analytical sensitivity experiments revealed high assay efficiency (> 100%) and sensitivity, with approximate 95% LOD 0.05–0.5 genome equivalents (1–10 fg/µL) per reaction for *T. brucei brucei* and 0.06–0.6 genome equivalents (1–10 fg/µL) per reaction for *T. congolense* Savannah (S1). Analytical specificity testing of TBR-qPCR revealed successful amplification in 9/9 target DNA samples (*T. brucei* M249, *T. b. rhodesiense* Z212, *T. b. gambiense* ELIANE in triplicate). However, low-level (Cq > 35) amplification was also recorded in non-target *Trypanosoma* DNA samples; 3/3 *T. godfreyi*, 2/3 *T. simiae* and 1/3 *T. congolense* Kilifi (S2). TCS-qPCR analytical specificity testing revealed amplification in 3/3 target DNA samples (*T. congolense* IL3000 in triplicate). However, some amplification was also recorded in non-target *Trypanosoma* DNA samples; 3/3 *T. simiae*, 3/3 *T. congolense* Forest and 2/3 *T. simiae* Tsavo (S2).

### DNA extraction efficiency

Extracted DNA from faecal samples collected across both studies had average DNA concentration of 52.0 ng/µL ± 1.01 standard error (SE). The mean A_260/A280_ purity ratio across all samples was 2.0 ± 0.01 SE which is greater than the expected optimal (1.80). However, the mean A_260/230_ ratio of 1.5 ± 0.02 SE was considerably lower than the expected range (2.00–2.20), indicating potential presence of contaminants.

Detection of sample processing control (SPC) in all faecal samples collected across the *T. congolense* Savannah infection study (n = 151) using a proprietary probe-based assay (Eurogentec, Seraing, Belgium) revealed successful target amplification in 100% of samples; one sample was removed from analysis due to processing error. Mean Cq was 28.82 ± 0.109 SE.

### Detection of T. brucei DNA in infected cattle samples

A total of 146 individual faecal samples were collected, 12 pre-inoculation and 134 post-inoculation. Of the post-inoculation samples, 68.7% (n = 92) could be linked to individual calves. Faecal samples were collected at seven days and three days before inoculation, then subsequently between three days post-inoculation and 68 days post-inoculation over 31 discrete sampling days. No visible blood was recorded in any faecal samples upon collection.

**Figure 1:**
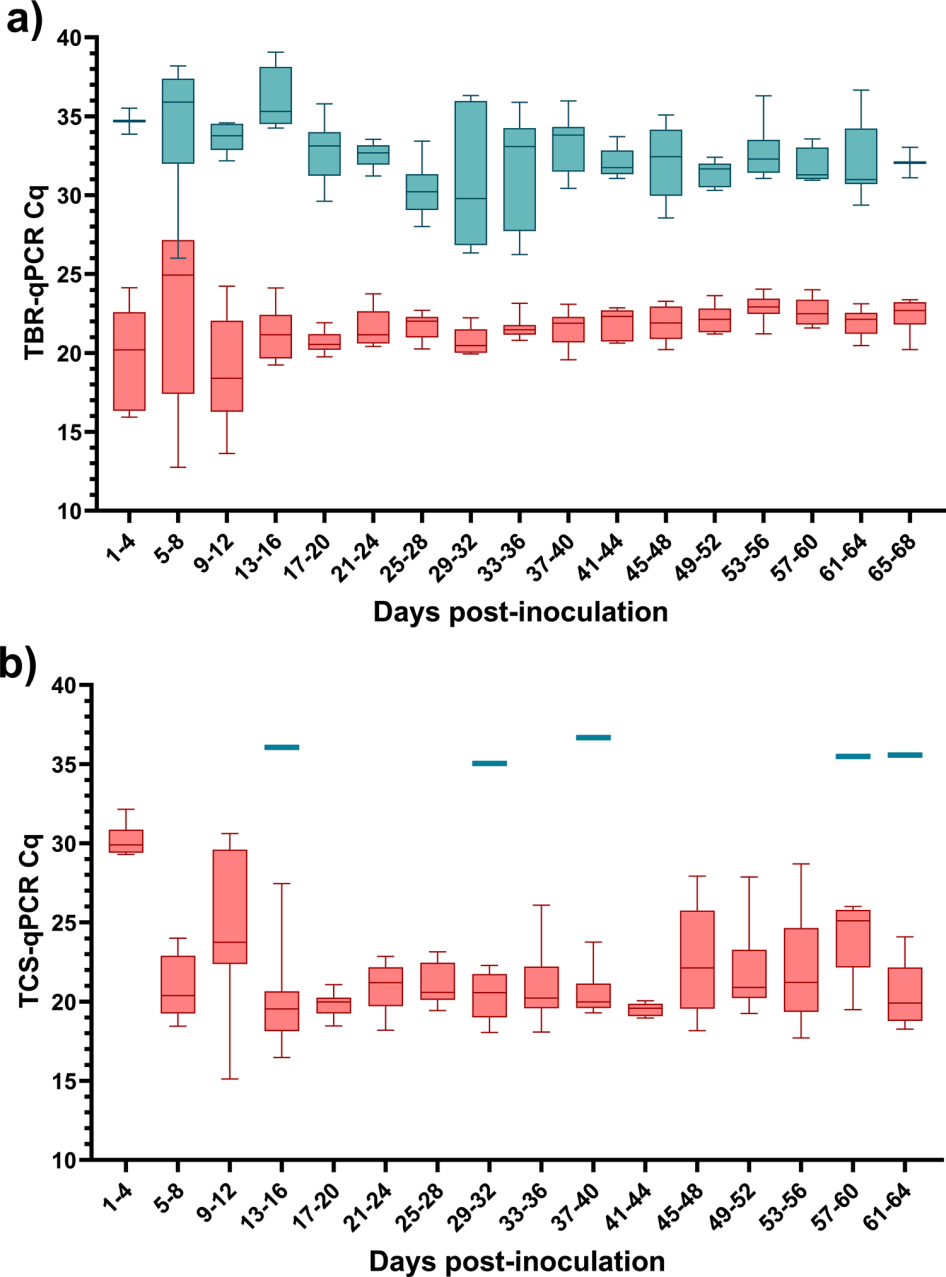
Box plot charts displaying Cq values obtained from screening all blood (red) and all faecal (blue) samples (including those of unknown calf origin) with TBR-qPCR (**a**) and TCS-qPCR (**b**) over consecutive four-day interval periods. Error bars represent range. Four-day intervals were chosen as this represented the smallest interval over which faecal and blood samples could be grouped and which also equally divided the study length (68 days).

Target DNA was successfully detected in 85% (n = 114) of post-inoculation faecal samples by TBR-qPCR and 50% (n = 67) by TBR-PCR (Fig. 1a). Target DNA was detected in faecal samples collected between 4 days post-inoculation to 66 days post-inoculation by both TBR-qPCR and TBR-PCR. Target DNA was detected in blood samples between 3 and 68 days post-inoculation. Presence of *T. brucei* target DNA was confirmed by the presence of a 173 bp TBR-PCR amplicon band visible by electrophoresis (S3). In post-inoculation blood samples, target DNA was detected in 100% (n = 138) of samples by TBR-qPCR. No amplification was recorded in pre-inoculation blood (n = 10) or faecal (n = 12) samples by PCR or qPCR.

Additionally, a sub-set of matched post-inoculation faecal and blood samples (n = 81) were screened for the presence of a *T. brucei* single-copy genomic target using the PLC-qPCR assay. PLC target DNA was detected in 2.47% (n = 2/81) of faecal samples and 97.53% (n = 79/81) of blood samples using a Cq cut-off of 37 (Fig. 3). Melt analysis revealed a target amplicon melt temperature between 80.08 and 83.38 °C across all samples.

### Confirmation of T. brucei DNA amplification

Sequencing of TBR-PCR 173 bp target products revealed high homology to *T. brucei* satellite DNA target entry (accession number K00392.1). Across forward and reverse sequences obtained from four different faecal samples, BLAST analysis revealed average query cover of 93.38% (± 2.03 SE) and average percentage identity of 91.59% (± 1.00 SE). The variable homology is to be expected due to the heterogeneity of the target sequence^21,39^.

### Detection of T. brucei DNA in individual calf post-inoculation samples

**Figure 2:**
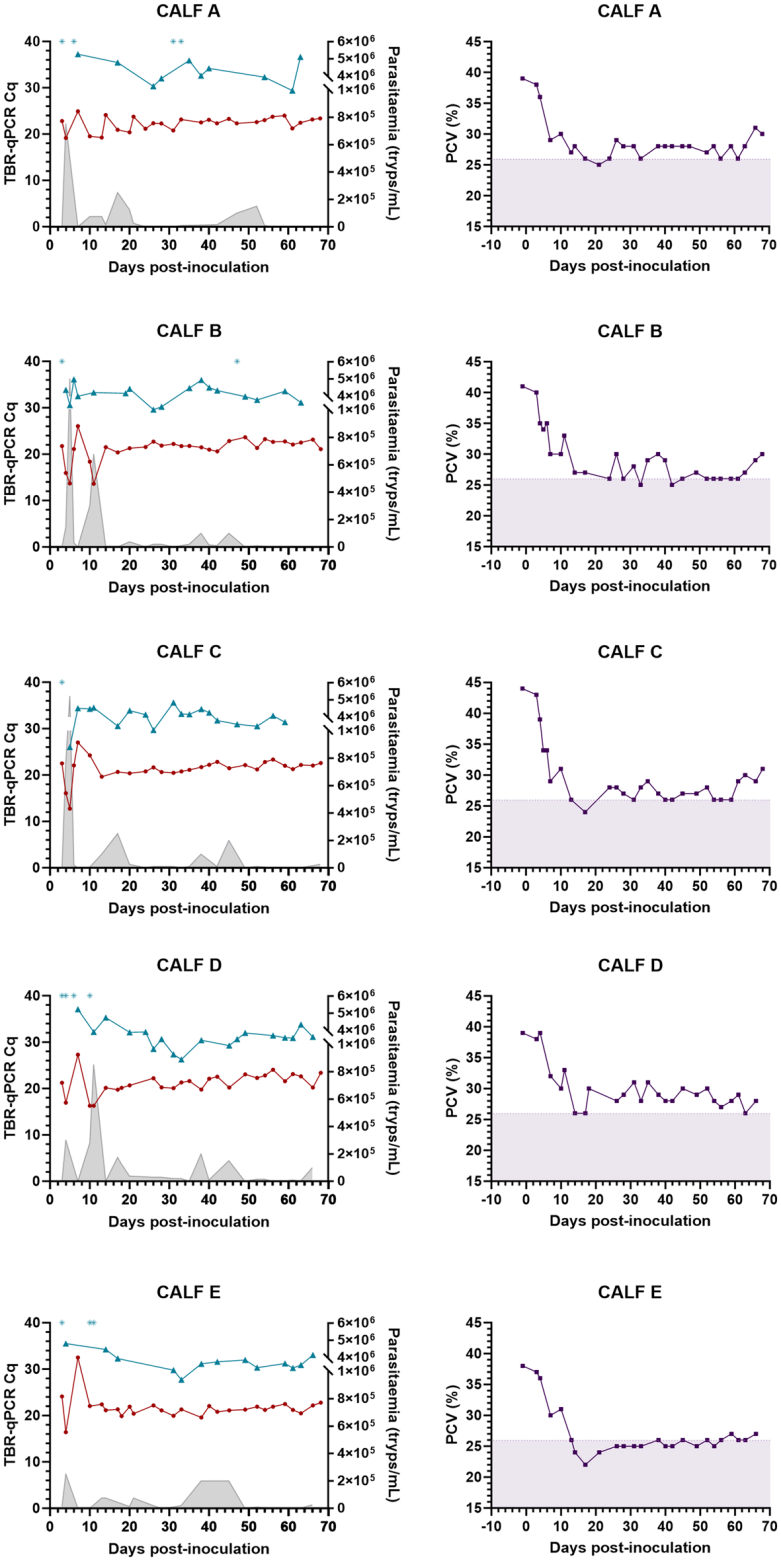
Plots displaying TBR-qPCR Cq values for post-inoculation blood and faecal samples, parasitaemia and PCV for each calf (A–E) over time. (left-hand plots) Parasitaemia values (shaded grey) are expressed in trypanosomes/mL (tryps/mL) of blood (right Y axes). Blood TBR-qPCR Cq values are shown as red circular symbols. Faecal TBR-qPCR Cq values are shown as blue triangular symbols. Faecal samples where TBR-qPCR recorded no amplification are marked with blue asterisk symbol at the top of each plot. (right-hand plots) PCV values are shown as purple square symbol. Shaded area represents the PCV range that would be considered abnormal or anaemic for a cow.

**Figure 3:**
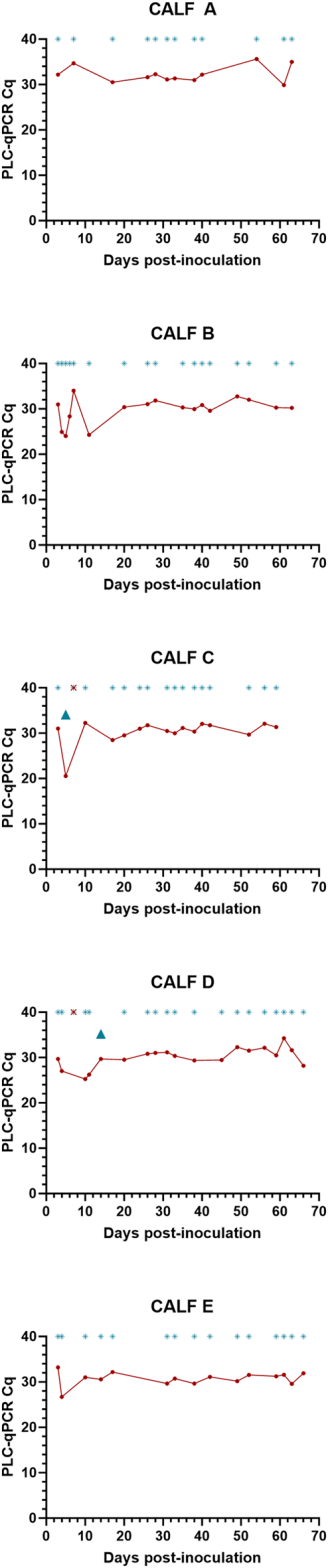
Plots displaying individual calf (A–E) PLC-qPCR Cq values for a subset of matched post-inoculation blood and faecal samples (n = 81). Blood PLC-qPCR Cq values are shown with red circular symbol. Blood samples where PLC-qPCR recorded no amplification are marked with a red asterisk at the top of each plot. Faecal PLC-qPCR Cq values are shown with a blue triangular symbol. Faecal samples where TCS-qPCR recorded no amplification are marked with a blue asterisk symbol at the top of each plot.

**Figure 4:**
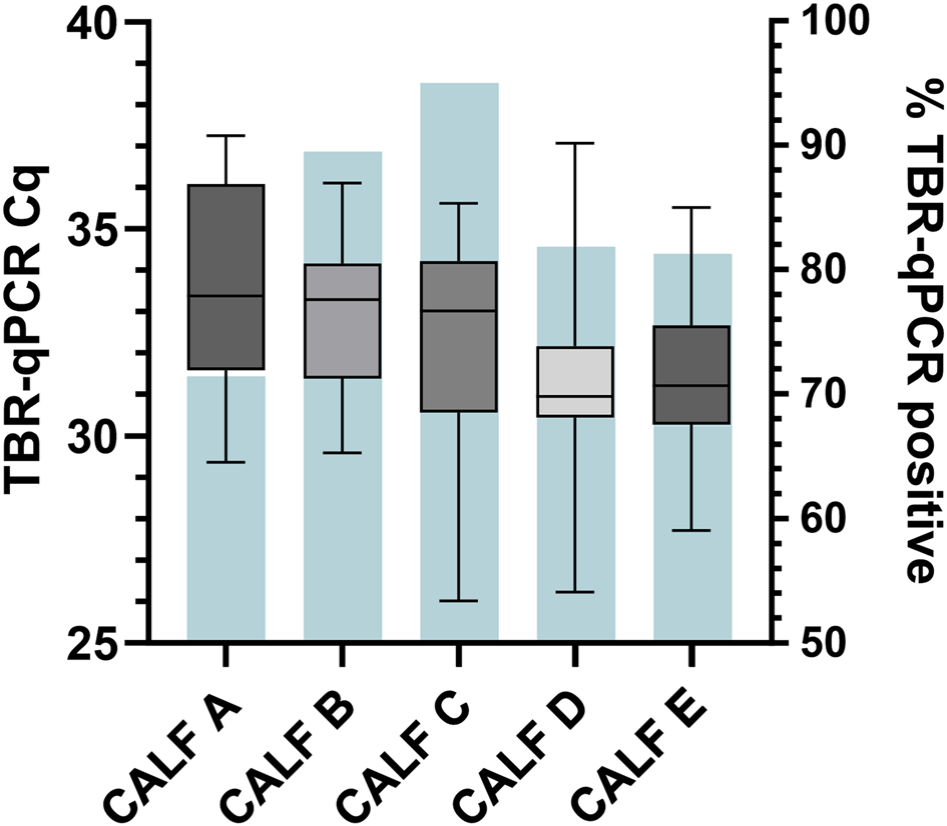
A box-and-whisker plot charting total faecal sample TBR-qPCR Cq values (left axis) attained for calves A (n = 10), B (n = 17), C (n = 19), D (n = 18) and E (n = 13). Horizontal line in box represents mean, error bars represent the minimum and maximum. Bar chart (blue, right axis) represents the proportion of total post-inoculation faecal samples for each calf that tested positive by TBR-qPCR.

Although calf parasitaemia varied between individuals, all had an initial parasitaemia peak (2.5 × 10^5^ to 5.2 × 10^6^ tryps/mL) and low blood TBR-qPCR Cq values (< 20) within 4–5 days post-inoculation, followed by rapid PCV decrease (Fig. 2). This was followed by smaller parasitaemia waves, yet relatively stable PCV and TBR-qPCR Cq values in blood and faecal samples across all calves for the remainder of the study (Fig. 2). This pattern was also seen across individual calf PLC-qPCR Cq values for a sub-set (n = 81) of matched faecal and blood samples (Fig. 3). For faecal samples recording amplification by TBR-qPCR (Cq < 40), there was a weak yet significant difference in total Cq values obtained from individual calves over the duration of the study (F[4,75] = 2.630, *p* = 0.041) (Fig. 4), however post-hoc Tukey tests revealed no significant differences. Detection of *T. brucei* DNA in faeces by TBR-qPCR in calves A-E were as follows; 71.43% (n = 10/14), 89.47% (n = 17/19), 95% (n = 19/20), 81.82% (n = 18/22) and 81.25% (n = 13/16) (Fig. 4). There was no significant difference between total TBR-qPCR Cq values obtained from individual calf blood samples (F[4,76] = 0.948, *p* = 0.441).

### Quantification and relationship of T. brucei DNA in faecal and blood samples

Whilst the quantity of detected *T. brucei* DNA in the blood reflected parasite load and calf clinical outcome, there were no such relationships with the amount of *T. brucei* DNA in the faeces. Although there were statistically significant relationships between TBR-qPCR blood Cq values and (log transformed) parasitaemia values (Y = -1.940*X + 29.68, R^2^ = 0.4807, *p* =  < 0.0001) and PCV (Y = − 0.3595*X + 36.31, R^2^ = 0.05770, *p* = 0.0059), there was no statistically significant relationship between faecal Cq and (log transformed) parasitaemia values (*p* = 0.6866) nor with PCV (*p* = 0.4011).

As expected, the amount of *T. brucei* DNA detected in the blood was consistently higher than in matched faecal samples. TBR-qPCR Cq values obtained from faecal samples were consistently higher (mean = 32.54, ± 0.237 SE) than for blood samples (mean = 21.54, ± 0.201 SE; Fig. 1).

**Figure 5:**
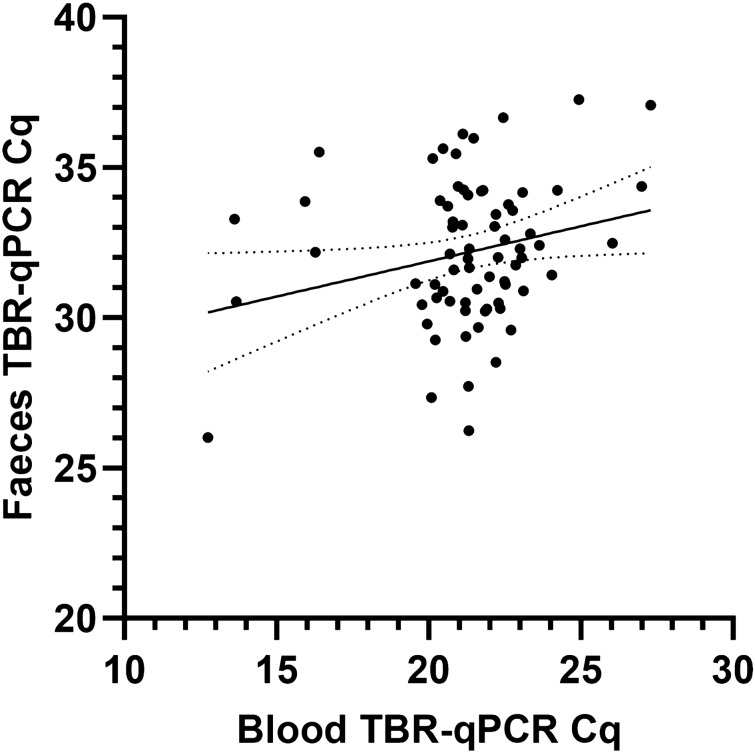
Linear regression analysis between individual calf matched blood and faecal TBR-qPCR Cq values (n = 70). Y = 0.2339*X + 27.20, R^2^ = 0.06089*p* = 0.0380. Solid line represents line of best fit and dotted lines represent 95% confidence bands.

Linear regression analysis revealed a weak yet statistically significant positive relationship (p = 0.0354, R^2^ = 0.06345) between Cq values obtained from matched blood and faecal samples (Fig. 5). The lowest Cq values (faecal = Cq 26.01, blood = Cq 12.75) were obtained from samples taken from the same calf at 5 days post-inoculation, coinciding with the first parasitaemia peak.

As expected, there was strong agreement between matched Cq values obtained from TBR-qPCR and PLC-qPCR screening of blood samples, with linear regression showing a strong statistically significant positive relationship (Y = 0.9303*X + 10.81, R^2^ = 0.8320, *p* =  < 0.0001).

### Factors associated with T. brucei DNA detection in infected cattle faeces

Although clinical factors (parasitaemia and PCV) were not associated with the amount of *T. brucei* DNA in faeces, faecal TBR-qPCR detection rate did increase over the course of the infection study. Whereas faecal samples collected during the first two weeks of infection had a detection rate of 61.11% (n = 22/36) by qPCR and 19.44% (n = 7/36) by PCR, faecal samples collected during weeks 4–10 had overall detection rate of 93.88% (n = 92/98) by qPCR and 61.22% (n = 60/98) by PCR. There was a moderate yet statistically significant negative association between time since inoculation (days) and faecal sample TBR-qPCR Cq value (Y = -0.03586*X + 33.75, R^2^ = 0.06741, *p* = 0.0053). These analyses suggest that where DNA was detected in faeces, the amount of this DNA generally increased over time (Fig. 2). There was no association between TBR-qPCR Cq value and sample DNA concentration (Y = 0.01208*X + 31.86, *p* = 0.355), showing that the amount of *T. brucei* DNA detected in faeces was independent of total quantity of DNA extracted from each sample.

### Detection of T. congolense DNA in infected cattle samples

A total of 151 individual faecal samples were collected, 6 pre-inoculation and 145 post-inoculation. Of the post-inoculation faecal samples, 100% could be linked to individual calves. In addition, a total of 167 blood samples were collected, 12 pre-inoculation and 155 post-inoculation. Faecal samples were collected at two days before inoculation, then subsequently on the day of inoculation through to 63 days post-inoculation over 25 discrete sampling days. No visible blood was recorded in any faecal samples upon collection.

**Figure 6:**
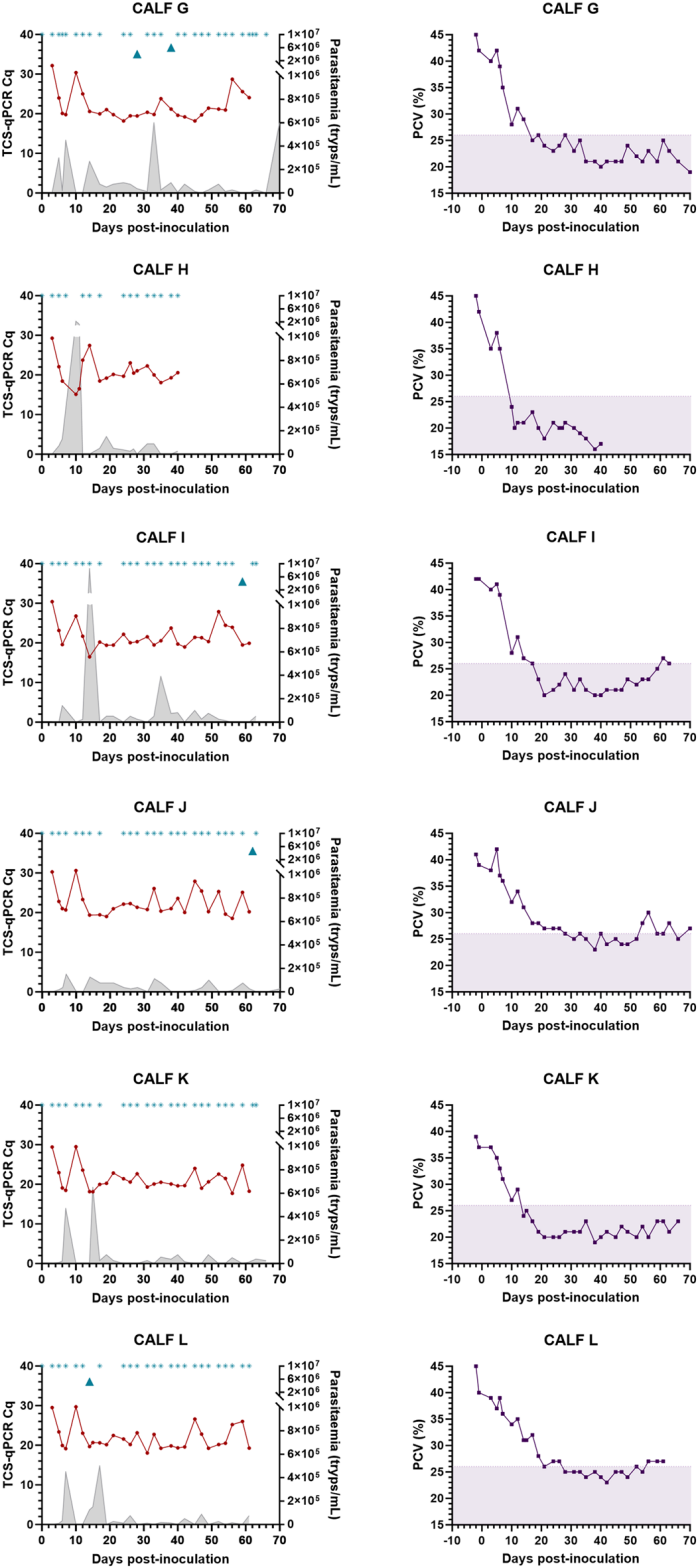
Plots displaying individual calf (G-L) TCS-qPCR Cq values for post-inoculation blood and faecal samples, parasitaemia and PCV over time. (left-hand plots) Parasitaemia values (shaded grey) is expressed in trypanosomes/mL (tryps/mL) of blood (right Y axes). Blood TCS-qPCR Cq values are shown as red circular symbols. Faecal TCS-qPCR Cq values are shown as blue triangular symbols. Faecal samples where TCS-qPCR recorded no amplification are marked with a blue asterisk symbol at the top of each plot. (right-hand plots). PCV values are shown as purple square symbols. Shaded area represents the PCV range that would be considered abnormal or anaemic for a cow.

Target DNA was detected in only 3% (5/145) of post-inoculation faecal samples by qPCR (mean = 35.76, ± 0.277 SE) and none by PCR (Figs. 1 and 6). Target DNA was detected in faecal samples collected at 14, 28, 38, 59 and 62 days post-inoculation. However, *T. congolense* DNA was detected in 100% (n = 155) of post-inoculation blood samples by TCS-qPCR (mean = 21.72, ± 0.260 SE; Fig. 6). No amplification was recorded in pre-inoculation blood (n = 12) or faecal (n = 6) samples by PCR or qPCR.

### Detection of T. congolense DNA in individual calf post-inoculation samples

Similar to the *T. brucei* infection study, all calves experienced a parasitaemia peak (1.4 × 10^5^ to 2.2 × 10^6^ tryps/mL), low TCS-qPCR Cq values (< 21) and PCV decrease (11.5% average) within the first 5–10 days of the infection, followed by more variable parasitaemia and TCS-qPCR Cq values for the duration of the study (Fig. 6). One-way ANOVA analysis determined there to be no significant difference between total mean blood Cq values obtained from calves G–L (F[5,149] = 0.796, *p* = 0.5542). *T. congolense* DNA was detected in the faeces of four calves (calves G, I, J, L). Parasitaemia varied throughout the course of the infection, however the first parasitaemia peak appeared between 5 and 10 days post-inoculation across all calves.

### Quantification and relationship of T. congolense DNA in blood samples

As anticipated, the detection of *T. congolense* DNA in the blood was strongly associated with parasitaemia and PCV. Linear regression analyses revealed a statistically significant negative relationship between matched TCS-qPCR blood Cq values and (log transformed) parasitaemia values (Y = -0.6265X + 23.64, R^2^ = 0.20, *p* < 0.0001) and a statistically significant positive relationship between blood Cq values and PCV values (Y = 0.5990X + 12.88, R^2^ = 0.1082, *p* < 0.0001).

## Discussion

These results establish, for the first time, the ability to consistently detect *T. b. brucei* DNA in the faeces of infected cattle. In contrast, *T. congolense* Savannah DNA was detected rarely in faeces, despite the respective qPCR assays having very similar approximate limits of detection (0.05–0.5 genome equivalents per reaction for TBR-qPCR and 0.06–0.6 genome equivalents per reaction for TCS-qPCR). This finding may be explained by the differences in *Trypanosoma* species ability to extravasate, and potentially consequent tissue distribution; with *T. brucei* capable of extravascular tissue and lymph invasion, whilst all evidence suggests *T. congolense* remains restricted to the blood circulatory system^4,5^. For *T. brucei,* the intestinal lumen presents the most obvious point of entry into the faeces. *T. brucei* has previously been recorded in extravascular lymphoid and adipose tissue surrounding the gastrointestinal tract (GIT) in mice^37,38^, and in peritoneal fluid of infected sheep^40^—however, there was no evidence of extravascular *T. congolense* in similar infection studies in rats^41^. To what extent *T. brucei* invades GIT mucosal or epithelial tissue, or sequesters in gut-associated lymphoid tissue within the intestines, is not certain. Direct haemorrhage of blood into the GIT lumen could also provide a viable route of entry of parasites/parasite DNA, and whilst intestinal damage is known to occur in *T. brucei* infection of mice^34^, no visible blood was recorded in any of the faecal samples collected in this study. However, presence of occult faecal blood could not be ruled out. Characterisation of the extent of GIT lesions in the cow as a result of *T. brucei* or *T. congolense* infections have not been reported in the literature to the best of our knowledge.

The negative association between time since inoculation and faecal TBR-qPCR Cq value demonstrates that, on average, the amount of *T. brucei* DNA detected in faeces increased over the course of the infection. Although higher concentrations of *T. brucei* DNA were generally detected in the later phases of the infection, the highest quantity of DNA in faeces was detected in a sample collected at 5 days post-inoculation (Fig. 2). A blood sample taken from the same calf (calf C) on the same day recorded the highest quantity of target DNA across all samples, with a high parasitaemia level of 5.2 × 10^6^ tryps/mL (Fig. 2). In fact, all calves had an initial parasitaemia peak within 4–5 days post-inoculation. These early *T. brucei* parasitaemia and ‘peaks’ of DNA detection agree with previous studies that found a *T. brucei* AnTat 1.1 parasitaemia peak within 4–5 days post-inoculation in weaned Holstein–Friesian calves^19,42^. Similarly, a study conducted by Doko et al.^43^ found a parasitaemia peak at 5–6 days post-inoculation in Borgou and Lagune cattle inoculated with *T. brucei* AnTat 1.1E. While this was earlier than the parasitaemia peaks observed by Van den Bossche et al. in Holstein cattle inoculated with *T. brucei* EATRO 1125 (an alias for AnTat 1.1), that experiment involved tsetse rather than needle challenge^44^. The weak yet statistically significant positive association between TBR-qPCR Cq values in matched blood and faecal samples indicates that whilst DNA quantity in faeces generally reflects quantity of DNA in the blood, it is a complex relationship that is impacted by factors beyond the scope of this simple linear regression. This is evidenced by the lack of significant relationships between amount of DNA in faeces with either parasitaemia or PCV. In contrast, strong statistically significant associations were found between amount of parasite DNA in the blood with both parasitaemia and PCV. This may be linked to the extravascular tissue distribution of *T. brucei*, which is an important question that remains to be properly characterised in cattle.

Whilst widespread use of qPCR for AAT clinical diagnostics remains financially unviable and logistically unfeasible, it is worth discussing the potential utility of faecal screening in this context. While anaemia has long been considered a key diagnostic indicator of trypanosome infections in the field, in the infection study described here, *T. brucei* DNA remained detectable in faeces even when calves recorded PCV within the ‘normal’ range (26–45%) (Fig. 2)—indeed 38.6% of TBR-qPCR positive faecal samples originated from animals with PCV > 26%. The ability to detect an active infection in animals without overt anaemia suggests that faecal qPCR screening could offer a more sensitive and accurate indication of active infection than diagnosis made on presentation of anaemia alone, which could be important in both treatment decisions and in surveillance efforts to gauge infection prevalence (e.g., stage 1 of the Progressive Control Pathway for AAT^45^). However, it is not known for how long *T. brucei* DNA continues to be shed in faeces if and when infection is cleared—this could be tested, for example, following curative drug treatment in experimental infections.

The ability to detect the single-copy *Trypanozoon* glycosylphosphatidylinositol-specific phospholipase C (GPI-PLC) gene in faecal samples is a key finding. Aside from being a primary cause of AAT, *T. brucei* subspecies *T. b. rhodesiense* and *T. b. gambiense* are the cause of HAT. Faecal screening—either in livestock or wildlife—could potentially be utilised in Rhodesian HAT (rHAT) surveillance. As a zoonotic disease with both wildlife and livestock reservoirs, where parasite distribution is particularly difficult to predict or quantify. Wildlife surveillance usually relies on opportunistic blood sampling with veterinary intervention. However, faecal sampling could broaden the scope of both the species surveyed and the sampling scale. Currently, molecular confirmation of rHAT DNA relies on the detection of a single-copy gene target—the serum resistance-associated (SRA) gene^24^. The *Trypanozoon*-specific GPI-PLC gene is also single-copy and is used in molecular assays to differentiate *T. brucei* sub-species^46,47^. In the current study, GPI-PLC detection by PLC-qPCR was used as a proxy for SRA detection (as *T. brucei* AnTat 1.1 does not possess the SRA gene). Although the GPI-PLC target was detected in only 2/81 of the faecal sample sub-set, it shows the potential of using this technique to detect *T. b. rhodesiense* DNA in wildlife samples. However, sensitivity remains a major obstacle, and whilst further DNA extraction optimisation and assay development may improve the detection rate, there are several other environmental factors (such as desiccation and UV exposure) to take into consideration when applying this method outside of the laboratory.

It is difficult to predict how or if the results of this study would change in field samples. Holstein–Friesian and other European taurine cattle breeds are highly susceptible to AAT, particularly in a study setting where animals are immunologically naïve and receive a large parasite inoculation intravenously. Many native cattle breeds and cross-breeds display a level of trypanotolerance leading to suppressed parasitaemia levels^48–50^ and therefore potential for decreased DNA shedding in faeces. Similarly, wildlife reservoir hosts such as Cape buffalo (*Syncerus caffer*) are also able to control and suppress parasitaemia^51,52^. Nonetheless it should be noted that in the current study, calf *T. brucei* parasitaemia tended to remain low or undetectable from 30 days post-inoculation (Fig. 2), indicative of chronic infection stage and consistent with infection profile of adult cattle in the field^9^. Therefore, the consistent detection of *T. brucei* DNA in the current study may well be reflective of field results. Of the two faecal samples testing PLC-qPCR positive, one was collected from a calf on the day of a parasitaemia peak and the other was taken from a calf that had no visible parasitaemia on the day of sampling, yet had experienced a parasitaemia peak three days previously. The ability to detect single-copy *T. brucei* targets in the faeces of low-parasitaemia hosts is critical to HAT surveillance, yet was not demonstrated consistently in the current study. Conversely, in a field setting these animals are also more likely to be co-infected with GIT pathogens such as helminths^53–55^, some species of which are likely to lead to blood directly entering the lumen, therefore increasing potential for faecal DNA shedding of *Trypanosoma*. Additionally, different *T. brucei* and *T. congolense* strains are known to vary in virulence^3,56^, likely also impacting the parasitaemia profile. It is currently not known how these complex host and parasite factors influence *Trypanosoma* DNA in faeces, and therefore field trials are necessary to investigate further.

Use of faecal screening in the field not only has to overcome qPCR assay hurdles; the sample itself presents obstacles. RNALater was successfully used as a DNA preserving agent in this study and enables samples to be stored at room temperature for seven days—theoretically a field-friendly technique. This, paired with an extraction kit designed to remove faecal inhibitors, resulted in extracted DNA of reasonable yield and quality that was comparable to the range found in other studies^57^. The low A_260/230_ ratio (1.54) is likely due residual carbohydrates and phenols in the eluted DNA, probably due to dietary plant fibre^58^. Further environmental factors that may degrade DNA in a field setting were outside the scope of this study, but include the effects of desiccation, temperature, UV exposure and effects of dung fauna on faecal samples. *T. brucei* DNA was detected in 85.71% (n = 36/42) faecal samples collected from the environment and therefore could not be attributed to a particular calf. Of these faecal samples, 28 were described as cold, dry, or disrupted upon collection. Whilst it is difficult to determine the timeframes based on these subjective observations, these results at least demonstrate the potential of using environmental faecal samples to detect *Trypanosoma* DNA. This would be particularly beneficial for wildlife host screening, where direct sampling is costly and logistically difficult. The approximate cost of collecting, processing and screening each faecal sample in this study was £5.83, most of which can be attributed to the DNA extraction kits used (Quick-DNA Fecal/Soil Microbe DNA Miniprep kit—Zymo Research Europe GmbH, Freiburg, Germany) at £4.90 per sample. Whilst sampling scale-up would reduce this cost, further DNA extraction optimisation should be explored using high-throughput methods or sample pooling to improve the economic viability of this epidemiological research tool. Whilst a sample processing control was used in this study, an internal host control target would be more beneficial in a diagnostic context. Amplification of cattle DNA from epithelial tissue present on the surface of faecal samples has previously been demonstrated^59^ and may be suitable for this purpose.

Despite receiving the same dosage of *T. congolense* Savannah IL3000 at inoculation, parasitaemia levels varied widely between individual calves throughout the course of the study (Fig. 6), as did the TCS-qPCR Cq values attained from blood samples (Fig. 6). As with the *T. brucei* study, amounts of *T. congolense* DNA in the blood reflected calf parasite load and PCV values. All calves recorded parasitaemia from at least six days post-inoculation, with initial parasitaemia peak between 5 and 10 days post-inoculation. This agrees with previous studies that found a *T. congolense* Savannah IL3000 parasitaemia peak at 5–7 days post-inoculation^42^ and *T. congolense* KONT2/133 parasitaemia peak at 4–8 days post-inoculation^19^ in weaned Holstein–Friesian calves. Similarly in adult Zebu cattle, Getahun et al., detected *T. congolense* parasitaemia peaks within the first 10 days of infection^60^, whilst Ikede et al., estimated the *T. congolense* Savannah parasitaemia peak to be 9.4 days post-inoculation^36^. Calf clinical outcomes were markedly worse than the *T. brucei* infection study, with all calves experiencing moderate to severe anaemia (PCV < 26%) throughout the course of the study (Fig. 6), and Calf H being euthanised at 42 days post-inoculation following persistently low PCV. These differences in clinical outcome are likely due to the known differences in pathogenesis between *T. brucei* and *T. congolense* Savannah infections, however strain virulence and individual calf immune factors likely played a role also^3,9^.

This study also describes the development and application of two new probe-based qPCR assays for detection of *Trypanozoon* and *T. congolense* Savannah DNA, respectively. Analytical sensitivity testing revealed approximate 95% LOD 0.05–0.5 genome equivalents (1–10 fg/µL) per reaction for *T. brucei brucei* and 0.06–0.6 genome equivalents (1–10 fg/µL) per reaction for *T. congolense* Savannah (S1). These LODs are comparable to other assays targeting TBR and TCS, including PCR^17^ and qPCR^20,21^. The sensitivity is also comparable to other more field-friendly *T. brucei* detection methods such as LAMP^61,62^ and recently developed CRISPR-based diagnostic tools^63^. However, analytical specificity testing revealed some low-level amplification in several non-target *Trypanosoma* species DNA samples (S2). Although previous assays targeting TBR and TCS have generally reported no non-target amplification, this aspect is difficult to evaluate due to lack of available full analytical specificity data^17,20,21,64^. In the case of TCS, Masiga et al.^17^, demonstrated no cross reaction with other *T. congolense* subspecies and Ahmed et al.^20^, reported no cross-reaction with *T. vivax*, however *T. simiae* was absent from these specificity panels. In-silico BLAST analyses of the primers designed in the current study did not reveal non-target sequence homology. However, there is a paucity of high-quality sequence entries for many *Trypanosoma* species such as *T. simiae, T. godfreyi* and *T. congolense* Kilifi and Forest. It was also recently reported that the TBR target region varies in copy number between *T. brucei* subspecies^21^, likely impacting assay sensitivity in wild-type isolates. Although suitable for the purposes of this closed-system experimental infection study, the TBR-qPCR and TCS-qPCR assays may require further optimisation or exploration of other *Trypanosoma* genomic targets (such as ITS) to reduce the risk of non-target amplification in other *Trypanosoma* species.

AAT is not the only blood-borne parasitic disease affecting livestock in sub-Saharan Africa. East Coast Fever (theileriosis), anaplasmosis and babesiosis are also leading causes of livestock morbidity and mortality across this region, alongside zoonoses such as brucellosis, Q fever, bovine tuberculosis, anthrax, antimicrobial resistant bacteria and fasciolosis. Whilst it is currently not known whether it is possible to detect the DNA of all of these pathogens in faeces, combining detection of such microorganisms alongside *T. brucei* in a panel or multiplex assay could boost the attractiveness and economic viability of this screening method.

In conclusion, whilst these findings show the potential of using faeces as an easily-accessible sample to screen for active *T. brucei* infection, blood sampling is still required to reliably detect *T. congolense* Savannah in cattle. Future research should focus on testing the utility of this novel diagnostic method in field and wildlife samples to broaden *T. brucei* (including *T. b. rhodesiense* and *T. b gambiense*) surveillance.

## Methods

### Ethics statement

Animal experiments were carried out in the Large Animal Research and Imaging Facility at the Roslin Institute, University of Edinburgh, under the auspices of United Kingdom Home Office Project License number PE854F3FC. Studies were approved by the Roslin Institute (University of Edinburgh) Animal Welfare and Ethical Review Board (study numbers L447 and L475). Care and maintenance of animals complied with University regulations and the Animals (Scientific Procedures) Act (1986; revised 2013) and with ARRIVE guidelines (https://arriveguidelines.org/).

### Sample collection

**Figure 7:**
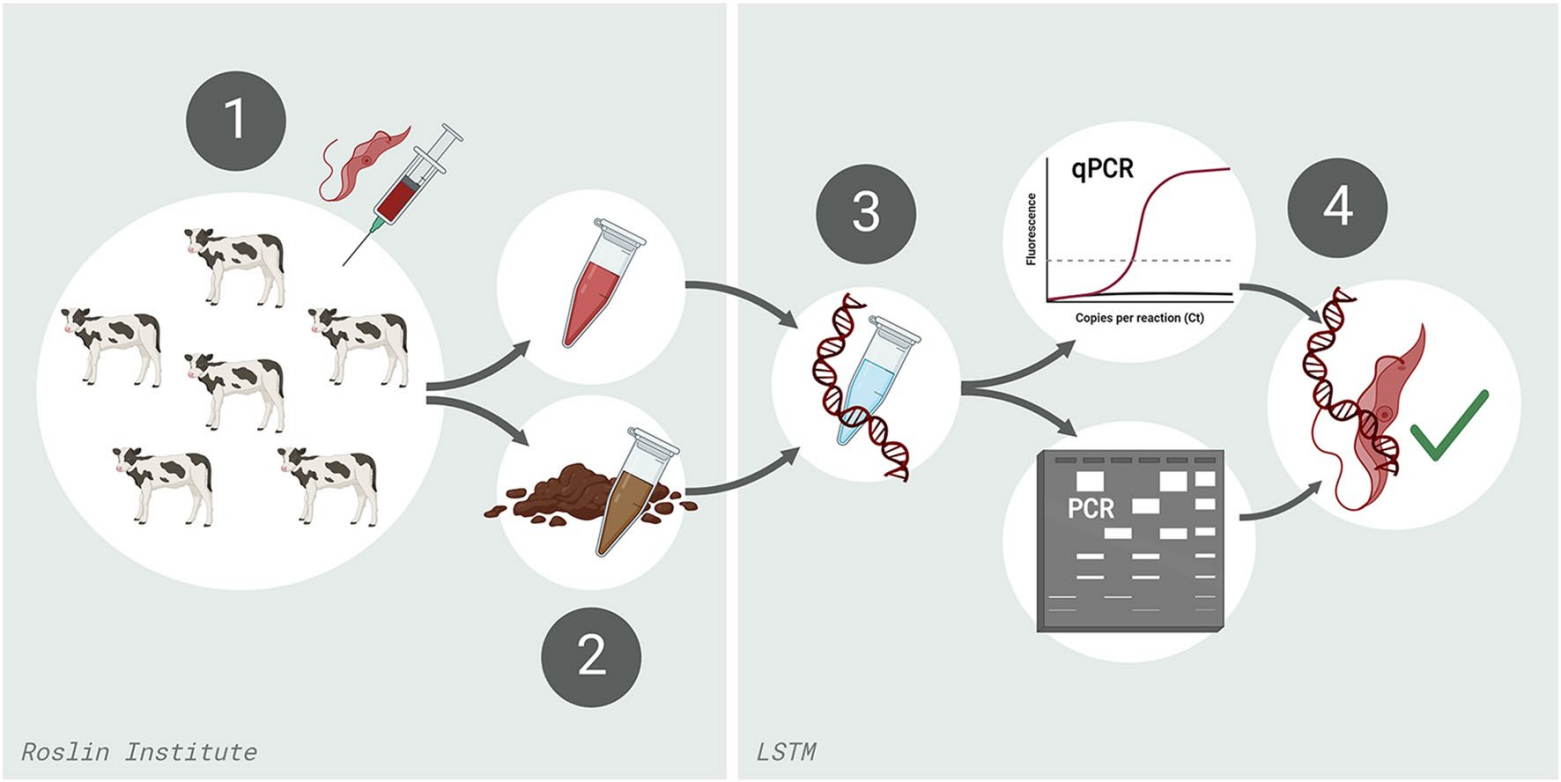
Study workflow. (1) Holstein–Friesian calves were inoculated with *Trypanosoma brucei brucei* AnTat 1.1 (n = 5) or *T. congolense* Savannah IL3000 (n = 6) in separate studies. (2) Blood samples and faecal samples (stored in RNA*later*®) were collected from each calf every 2–3 days for approximately 10 weeks. (3) All samples underwent DNA extraction before (4) screening with respective species-specific PCR and qPCR assays in order to detect and confirm the presence of *Trypanosoma sp.* DNA. Figure created with Biorender.com (www.biorender.com [accessed 15/11/2023]).

Experimental infections were carried out in vector-proof containment in the Large Animal Research and Imaging Facility at the Roslin Institute; male Holstein–Friesian cattle (n = 5 for *T. brucei* study, n = 6 for *T. congolense* study) of post-weaning age (4–6 months) were inoculated with 1 × 10^6^ trypanosomes (*T. brucei brucei* AnTat 1.1. or *T. congolense* Savannah IL3000) via the jugular vein (Fig. 7). Infections were followed for up to 68 days. Calf clinical signs were routinely monitored by rectal temperature and blood packed-cell volume (PCV). One animal (Calf H) was removed from the *T. congolense* study at 42 days post-inoculation due to persistently low PCV. Jugular blood samples were taken every 2–3 days for PCV and parasitaemia measurement, the latter using the quantitative buffy coat technique^65^. Faecal samples were collected post-defecation either from the floor, or following rectal temperature monitoring. Where possible, samples were taken from freshly deposited faeces that could be linked to individual calves. Approximately three grams of each faecal sample was added to a pre-prepared collection tube containing RNA*later* (Fig. 7). For each sample, a submission form was completed detailing sample ID, date and time of sample collection and whether blood was visible in the faecal matter. Where possible, calf ID was also recorded. Faecal samples in RNA*later* were stored at – 80 °C until further processing.

### DNA extraction

Faecal samples were processed using Quick-DNA Fecal/Soil Microbe DNA Miniprep kit (Zymo Research Europe GmbH, Freiburg, Germany). Approximately 150 µg of each thawed faecal sample was processed according to the manufacturer’s protocol. Additionally, to assess faecal sample DNA extraction and inhibition, a sample processing control (SPC) kit using Cy 5-QXL 670-labelled Taqman probe (Eurogentec, Seraing, Belgium) was included in the extraction of all faecal samples collected during the *T. congolense* infection study. In this instance 1 µL of SPC diluted 1:100 was added to each 150 mg faecal sample before being processed. Purified DNA was eluted in 100 µL elution buffer. The mechanical lysis step was performed using a TissueLyser II (QIAGEN, Hilden, Germany) with two rounds of lysis at 30 Hz for five minutes. Blood samples were processed using DNeasy 96 Blood and Tissue kits (QIAGEN, Hilden, Germany). 100 µL of each blood sample was processed according to the manufacturer’s protocol. Purified DNA was eluted in 100 µL elution buffer. All DNA samples were stored at – 20 °C until further use.

### DNA quantification, controls and preparation

DNA samples were quantified using an Implen NP80 spectrophotometer (Implen GmbH, Munich, Germany). Purified DNA from 12 different *Trypanosoma* species and sub-species were used as controls and in the analytical specificity panel (S4). All control DNA samples were diluted in TE to an approximate concentration of 1 ng/µL before use. Additionally, a composite sample encompassing equal parts DNA extracted from all pre-infection faecal samples was used as the negative faeces control (NFC).

### PCR

Primer sequences can be found in Table 1. All PCR reactions were carried out using MyTaq Red Mix (Meridian Bioscience, Cincinnati, USA) following the manufacturer’s protocol. Briefly, 5 µL of DNA template was added to 12.5 µL 2× MyTaq Red Mix, 0.5 µL forward and reverse primer and 6.5 µL nuclease-free water to give a 25 µL total reaction volume. Thermocycling conditions for TBR-PCR and TCS-PCR were as follows; 3 min at 95 °C initial denaturation, followed by 35 cycles of 15 s denaturation at 95 °C, 15 s annealing at 55 °C (TBR-PCR) or 60 °C (TCS-PCR) and 20 s extension at 72 °C, followed by a final extension for 2 min at 72 °C. Thermocycling was carried out using an Applied Biosystems Veriti thermal cycler (Life Technologies, Carlsbad, US). PCR products were separated by agarose gel electrophoresis and visualised using a UV transilluminator. *T. brucei* M249 or *T. congolense* GAM2 DNA at concentration of 1 ng/µL was used as the positive template control (PTC) for TBR-PCR assays and TCS-PCR assays, respectively. Nuclease-free water was used as the no-template control (NTC) for both assays.

**Table 1:** Oligonucleotide primers used in the study. SPC qPCR kit primer and probe sequences not given (Eurogentec, Seraing, Belgium).

Oligo name	Sequence (5′ → 3′)	Target	Assay Type	Source
TBR_PCR_F	CGAATGAATATTAAACAATGCGCAGT	*Trypanozoon* minichromosome satellite DNA repeat	PCR	^22^
TBR_PCR_R	AGAACCATTTATTAGCTTTGTTGC			
TCS_PCR_F	CGAGAACGGGCACTTTGCGA	*T. congolense Savannah* minichromosome satellite DNA repeat	PCR	^22^
TCS_PCR_R	GGACAAACAAATCCCGGGCACA			
TBR_QPCR_F	CGCAGTTAACGCTATTATACACA	*Trypanozoon* minichromosome satellite DNA repeat	qPCR	Current study
TBR_QPCR_R	CATTAAACACTAAAGAACAGCGT			
TBR_QPCR_PRB	FAM-TGTGCAACATTAAATACAAGTGTGT-ZEN			
TCS_QPCR_F	AACCACTATGCGCGTCAAAA	*T. congolense Savannah* minichromosome satellite DNA repeat	qPCR	Current study
TCS_QPCR_R	CACTTTGCGATTTTCCCAAA			
TCS_QPCR_PRB	HEX-CGTGCCAAATACGCGTTTTT-ZEN			
PLC1	CAGTGTTGCGCTTAAATCCA	*Trypanozoon* glycosylphosphatidylinositol-specific phospholipase-C gene	qPCR	^46,47^
PLC2	CCCGCCAATACTGACATCTT			

### qPCR assay development and reactions

For TBR-qPCR, the *T. brucei* sensu-lato minichromosomal satellite DNA entry (accession number K00392.1) was retrieved from GenBank and used as a template for primer design. For TCS-qPCR, six *T. congolense* Savannah minichromosomal satellite DNA sequences (accession numbers, JX910383.1, JX910382.1, JX910381.1, HE578914.1, X05769.1 and M30391.1) were retrieved from GenBank. Multiple sequence alignment was performed using Clustal Omega and analysed using Jalview v2. The resulting consensus sequence was used as the template for primer and probe design. All qPCR primers and probes were designed using Primer3. In silico primer sequence specificity analysis was performed using BLAST. qPCR primers used in the study are indicated in Table 1. All probe-based qPCR reactions were carried out using Bio-Rad SsoAdvanced Universal Probes Supermix (Bio-Rad Laboratories, Hercules, USA) following the manufacturer’s protocol. During optimisation of TBR-qPCR and TCS-qPCR, 5 µL of template DNA (1 ng/µL to 10 fg/µL) was mixed with 10 µL SsoAdvanced Universal Probes Supermix (2×), 250–400 nM forward and reverse primers, 150–200 nM probe, and nuclease-free water added to a 20 µL total reaction volume. For detection of the sample processing control (SPC), primers and probes were replaced with 10× Control Mix (Eurogentec, Seraing, Belgium) as per manufacturer’s protocol. Thermal cycling conditions during optimisation were as follows; initial denaturation at 95 °C for 3 min followed by 40 cycles of denaturation at 95 °C for 10 s and annealing and extension at 58–60 °C for 12 s (TBR-qPCR) or 30 s (TCS-qPCR). Data was captured during the annealing and extension step. All optimisation assays were carried out in triplicate. SYBR-based qPCR reactions were carried out using Agilent Brilliant III Ultra-Fast Master Mix (Agilent Technologies, Santa Clara, USA) following the manufacturer’s protocol. Briefly, 5 µL of template DNA was mixed with 10 µL Ultra-Fast Master Mix (2×), 200 nM of forward and reverse and primer and nuclease-free water to a total reaction volume of 20 µL. Thermal cycling conditions were as follows; initial denaturation at 95 °C for 3 min followed by 40 cycles of denaturation at 95 °C for 10 s and annealing and extension at 60 °C for 20 s. Data was captured during the annealing and extension step. Following cycling a melt step was performed between 65 and 95 °C at 0.3 °C per second. Thermocycling, fluorescence detection and data capture was carried out using a Mic and micPCR v.2.9.0 software (Bio Molecular Systems, Upper Coomera, Australia). *T. b. brucei* M249 at concentration of 1 ng/µL was used as PTC for the TBR-qPCR and PLC-qPCR assays, and *T. congolense* Savannah GAM2 DNA at 1 ng/µL was used for TCS-PCR assays. Nuclease-free water was used as NTC for all assays.

### PCR product sequencing

For TBR-PCR, 177 bp target products from four faecal samples (R44, R46, R80 and R84) and ~ 700 bp non-target products from four faecal samples were selected for sequencing analysis. Products were excised and purified using an Exo-CIP™ Rapid PCR Cleanup Kit (New England Biolabs, Ipswich, USA) following the manufacturer’s protocol. Resultant purified DNA was eluted in 20µL elution buffer. Sanger sequencing was performed by Source BioScience (Source BioScience Limited, Nottingham, UK) using both TBR_PCR_F and TBR_PCR_R primers (Table 1). Sequence clean-up and alignments were performed in BioEdit v7.2^66^. Resultant sequences were subject to BLAST nucleotide analysis (National Centre for Biotechnology Information).

### Statistical analysis

All data was collated into a centralised database in Excel (Microsoft). Further analyses and data visualisation were performed using SPSS Statistics v28 (IBM) and GraphPad Prism v10. Faecal data that could not be attributed to a particular calf (or matched to a blood sample) were excluded from individual calf data analyses and statistical analyses. All data are presented as the mean ± standard error (SE). One-way ANOVA followed by post-hoc Tukey tests were used to compare total results from different calves. Parasitaemia data were log-transformed prior to analysis to reduce data skew. Linear regression was used to test for associations between sample Cq values, time (days post-inoculation), PCV and parasitaemia. A *p* < 0.05 was considered significant.

### Supplementary Information

Supplementary Information.

## Data Availability

The datasets generated during and/or analysed during the current study are available under DOIs 10.6084/m9.figshare.24552466 and 10.6084/m9.figshare.24552508 at figshare.com.
