# Supplementary material for: Consistent detection of Trypanosoma brucei but not T. congolense DNA in faeces of experimentally infected cattle
Source: Sci Rep. 2024 Feb 20;14:4158. doi: 10.1038/s41598-024-54857-5 (PMC10879203; doi:10.1038/s41598-024-54857-5)
Supplement: Supplementary file 1 — Supplementary Information. [file 41598_2024_54857_MOESM1_ESM.pdf]

Consistent detection of *Trypanosoma brucei* but not *T. congolense* DNA in faeces of experimentally infected cattle

Saldanha, I.* [1], Betson, M.[2], Vrettou, C.[3], Paxton, E.[3], Nixon, J.[4], Tennant, P.[4], Ritchie, A.[4], Matthews, K.R.[5], Morrison, L.J.[3], Torr, S.J.[1], Cunningham, L.J[6].

[1]Liverpool School of Tropical Medicine, Vector Biology Dept.; [2]University of Surrey, School of Veterinary Medicine; [3]University of Edinburgh, Roslin Institute; [4]University of Edinburgh, Large Animal Research and Imaging Facility; [5]University of Edinburgh, Institute of Immunology and infection; [6]Liverpool School of Tropical Medicine, Dept. Tropical Disease Biology.

Isabel Saldanha*	isabel.saldanha@lstmed.ac.uk
Martha Betson	m.betson@surrey.ac.uk
Christina Vrettou	christina.vrettou@roslin.ed.ac.uk
Edith Paxton	edith.paxton@roslin.ed.ac.uk
James Nixon	James.Nixon@roslin.ed.ac.uk
Peter Tennant	peter.tennant@roslin.ed.ac.uk
Adrian Ritchie	aritchi3@exseed.ed.ac.uk
Keith R. Matthews	keith.matthews@ed.ac.uk
Liam J. Morrison	liam.morrison@roslin.ed.ac.uk
Stephen J. Torr	steve.torr@lstmed.ac.uk
Lucas J. Cunningham	lucas.cunningham@lstmed.ac.uk

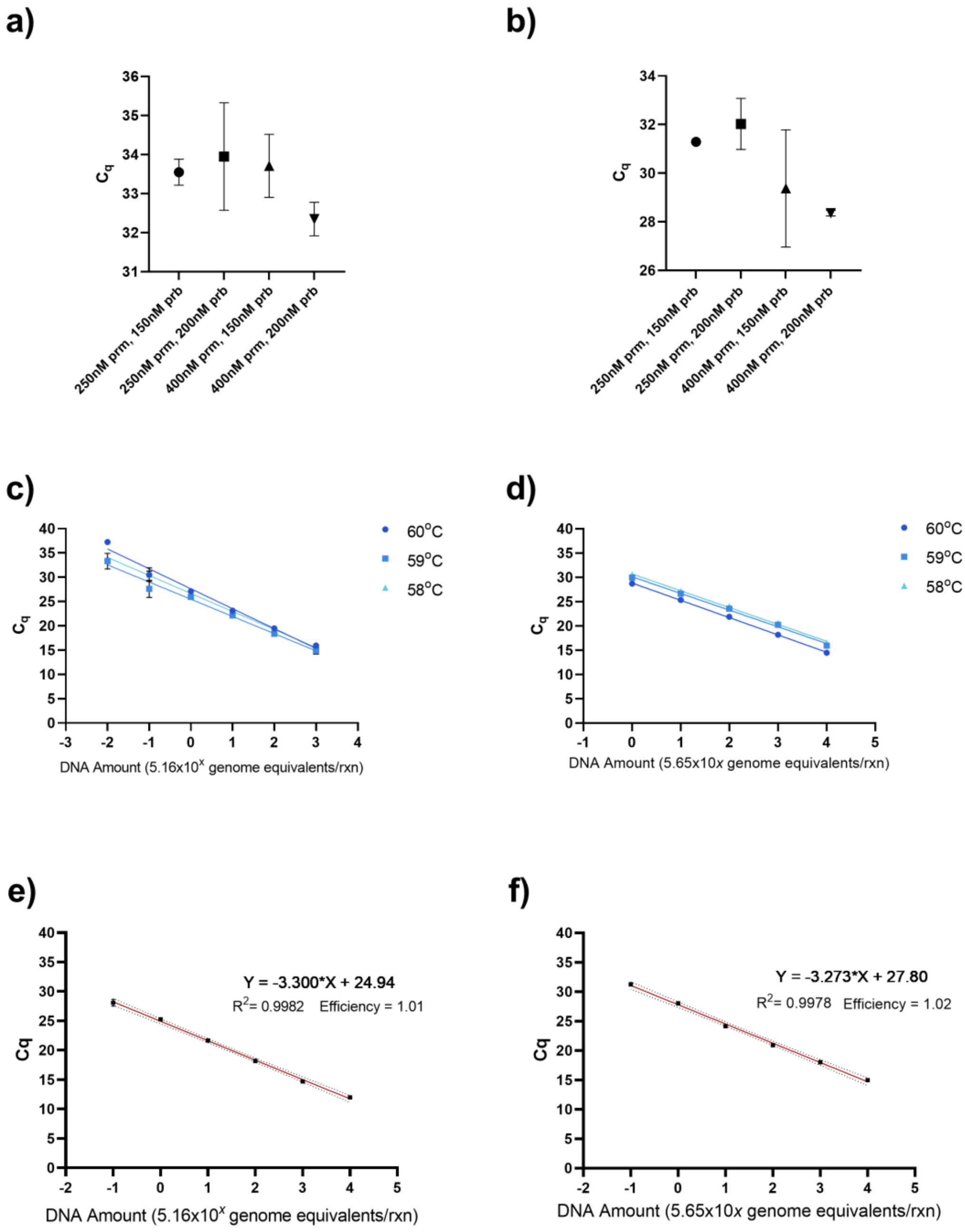

S1: (a-b) Charts displaying Cq values obtained from TBR-qPCR (a) and TCS-qPCR (b) screening against four different combinations of two primer and probe concentrations in 10fg/uL respective target DNA in triplicate. Symbols represent the mean, error bars represent the standard deviation. (c-d) Charts displaying standard curve analyses of TBR-qPCR (c) and TCS-qPCR (d) Cq values at different annealing temperatures (58°C, 59°C and 60°C) over ten-fold dilution series (1ng/µL to 10fg/µL) of respective target DNA. Assays performed in triplicate. Symbols represent the mean and error bars represent the standard deviation. (e-f) XY scatter plot with standard curve displaying performance of optimised TBR-qPCR (e) and TCS-qPCR (f) performance over respective detectable ranges in spiked dilution series. In short, 10 ng/µL target DNA of *T. brucei* AnTat 1.1 (e) or *T. congolense* IL3000 (f) was spiked into a composite pre-inoculation faecal DNA sample and subsequently serially diluted 1:10 across five further composite faecal DNA samples. Linear regression best-fit line shown in red with 95% confidence bands displayed as dotted lines.

a)

Species	Sub-species	Strain	DNA conc.	Target (Y/N)	Amplification (total = 3)	Mean Cq.
T. brucei	*brucei*	AnTat 1.1	1ng/µL	Y	3	16.84
T. brucei	*gambiense*	ELIANE	1ng/µL	Y	3	10.69
T. brucei	*rhodesiense*	Z3	1ng/µL	Y	3	20.67
T. congolense	Savannah	IL3000	1ng/µL	N	0	-
T. congolense	Forest	ANR3	1ng/µL	N	0	-
T. congolense	Kilifi	WG84	1ng/µL	N	1	38.06
T. godfreyi		Ken7	1ng/µL	N	3	36.36
T. simiae		TV008	1ng/µL	N	2	36.70
T. simiae	Tsavo	114	1ng/µL	N	0	-
T. vivax		Miranda	1ng/uL	N	0	-

b)

Species	Sub-species	Strain	DNA Conc.	Target (Y/N)	Amplification (total = 3)	Mean Cq.
T. brucei	*brucei*	AnTat 1.1	1ng/µL	N	0	-
T. brucei	*gambiense*	ELIANE	1ng/µL	N	0	-
T. brucei	*rhodesiense*	Z3	1ng/µL	N	0	-
T. congolense	Savannah	IL3000	1ng/µL	Y	3	14.96
T. congolense	Forest	ANR3	1ng/µL	N	3	33.19
T. congolense	Kilifi	WG84	1ng/µL	N	0	-
T. godfreyi		Ken7	1ng/µL	N	0	-
T. simiae		TV008	1ng/µL	N	3	16.70
T. simiae	Tsavo	114	1ng/µL	N	2	36.89
T. vivax		Miranda	1ng/µL	N	0	-

S2: Tables displaying analytical specificity results of optimised TBR-qPCR (a) and TCS-qPCR (b) assays. (a) TBR-qPCR analytical specificity testing was carried out against a panel of 12 *Trypanosoma sp.* DNA samples screened in triplicate. Testing revealed successful amplification in 9/9 target DNA samples (*T. brucei* M249, *T. b. rhodesiense* Z212, *T. b. gambiense* ELIANE in triplicate). However, low-level amplification was also recorded in non-target *Trypanosoma* DNA samples (3/3 *T. godfreyi*, 2/3 *T. simiae* and 1/3 *T. congolense* Kilifi. (b) TCS-qPCR analytical specificity testing revealed amplification in 3/3 target DNA samples (*T. congolense* IL3000 in triplicate). However amplification was also recorded in non-target *Trypanosoma* DNA samples; 3/3 *T. simiae* DNA samples, along with low-level amplification in 3/3 *T. congolense* Forest and 2/3 *T. simiae* Tsavo DNA samples.

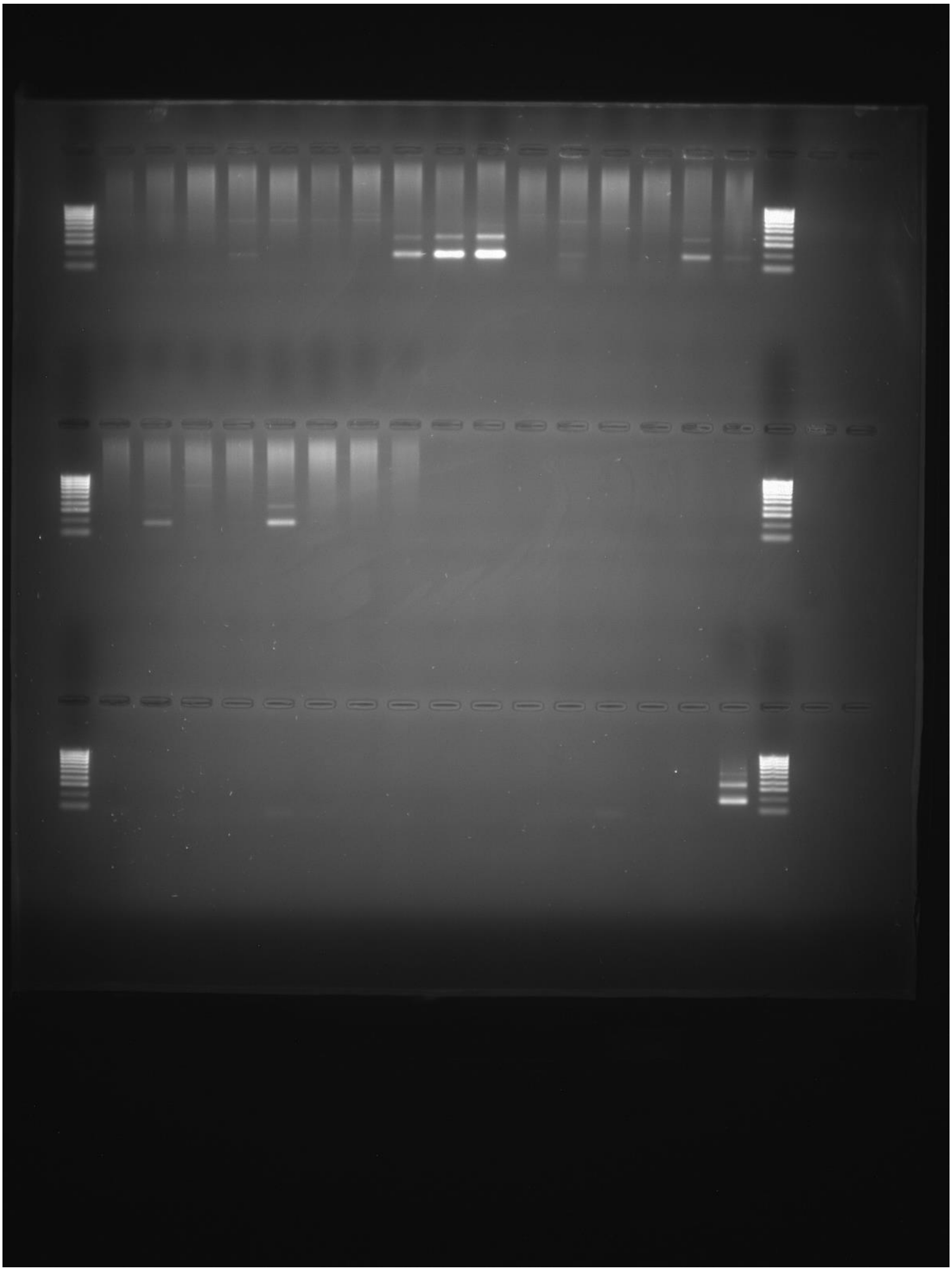

S3a: An unannotated and uncropped gel image displaying results of products resulting from TBR-PCR screening of DNA extracted from the first 23 post-inoculation cattle faecal samples. 100bp molecular ladder is present in wells 1 and 18 on each row.

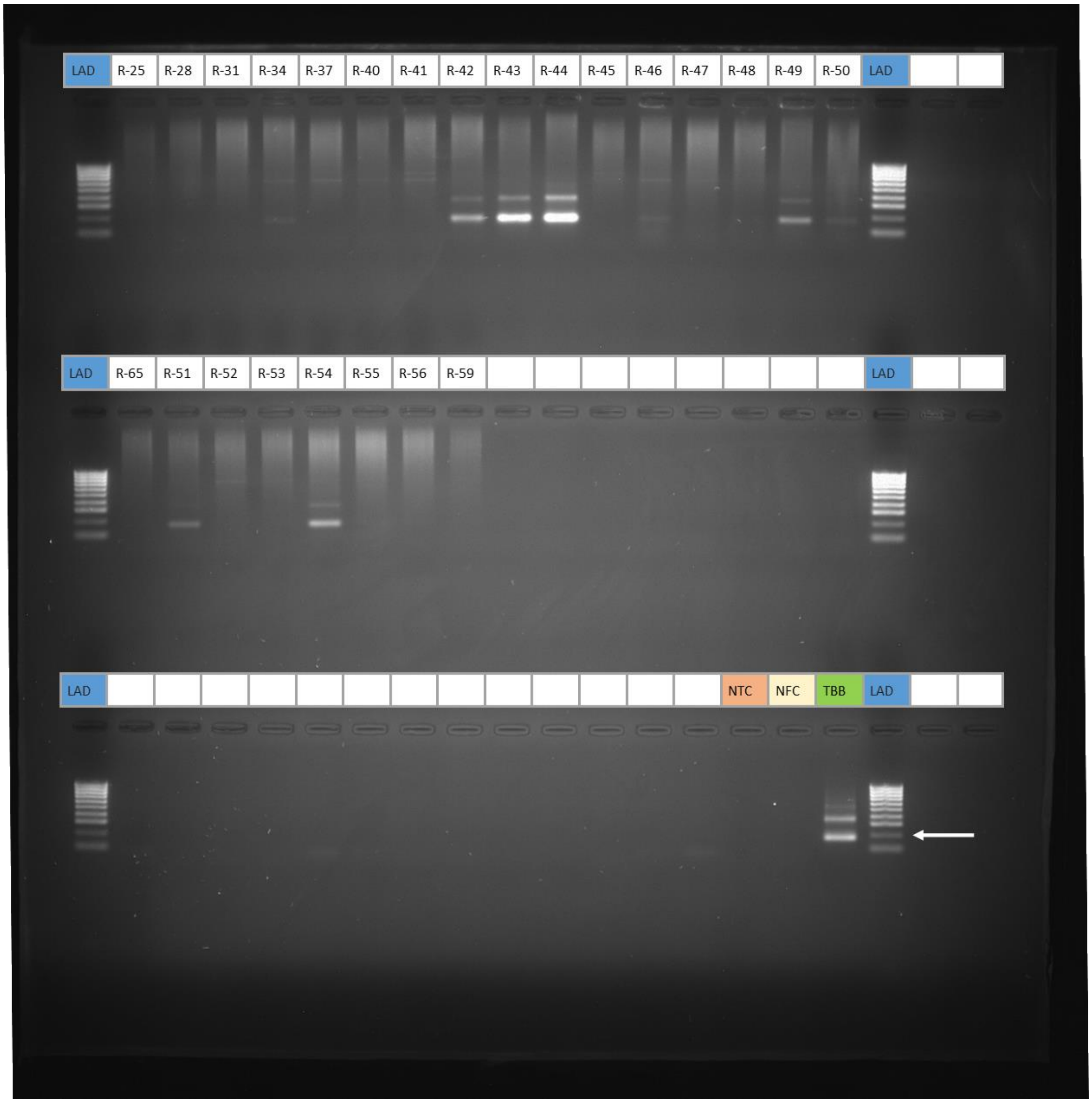

S3b: An annotated, rotated and cropped version of S3a gel image displaying results of products resulting from TBR-PCR screening of DNA extracted from the first 23 post-inoculation cattle faecal samples (three to ten days post-innoculation). LAD = 100bp molecular ladder, NTC = no-template control (nuclease-free water), NFC = negative faeces control. TBB = *T. b. brucei* M249 DNA 1 ng/µL (positive control). A white arrow in the lower right corner indicates the position of diagnostic 173 bp TBR-PCR target product, visible in positive control in addition to several samples.

**Species**	**Sub-species**	**Strain**	**DNA Concentration**
Trypanosoma brucei	*brucei*	M249	1ng/µL
Trypanosoma brucei	*brucei*	AnTat 1.1	1ng/µL
Trypanosoma brucei	*gambiense*	ELIANE	1ng/µL
Trypanosoma brucei	*rhodesiense*	Z4	1ng/µL
Trypanosoma congolense	Savannah	IL3000	1ng/µL
Trypanosoma congolense	Savannah	GAM2	1ng/µL
Trypanosoma congolense	Forest	ANR3	1ng/µL
Trypanosoma congolense	Kilifi	WG84	1ng/µL
Trypanosoma godfreyi		Ken7	1ng/µL
Trypanosoma simiae		TV008	1ng/µL
Trypanosoma simiae	Tsavo	114	1ng/µL
Trypanosoma vivax		Y486	1ng/µL
Trypanosoma vivax		IL1392	1ng/µL
Trypanosoma vivax		Miranda	1ng/uL

S4: *Trypanosoma sp.* DNA controls used in study.
